# Splenic injury severity, not admission hemodynamics, predicts need for surgery in pediatric blunt splenic trauma

**DOI:** 10.1186/s13037-019-0218-0

**Published:** 2020-01-03

**Authors:** Michel Teuben, Roy Spijkerman, Henrik Teuber, Roman Pfeifer, Hans-Christoph Pape, William Kramer, Luke Leenen

**Affiliations:** 1 Department of Trauma, University Medical Centre Utrecht, Suite G04.228, Heidelberglaan 100, 3585 GA Utrecht, The Netherlands; 2 Department of Trauma, University Hospital Zurich, Zurich, Switzerland; 3 Department of Pediatric Surgery, University Medical Centre Utrecht/ Wilhelmina Children’s Hospital Utrecht, Utrecht, The Netherlands

## Introduction

The spleen is the most frequently injured organ in blunt abdominal trauma and blunt abdominal trauma is a frequent cause of childhood injuries [1, 2]. The management of blunt splenic injuries (BSI) in pediatric patients has evolved over the past decades from primarily operative management to selective nonoperative management (NOM) [3–5]. In 1952, King and Schumacker were the first who described that splenectomised children were at risk for life-threatening infections [6]. Following reports that established the existence of a syndrome of overwhelming post splenectomy infection (OPSI), have eventually initiated the willing to preserve splenic function after trauma [7, 8]. Asplenic patients have a life time risk exceeding 5% on OPSI and this sepsis-syndrome has a mortality rate ranging between 50 and 80% [9–11].

While there are multiple advantages to selective NOM, there are also potential drawbacks. Failure of NOM (fNOM), defined as the need for surgical intervention in patients that were initially selected for conservative management, is associated with increased morbidity and mortality [12–14].

Selective nonoperative management has proven highly successful, and success rates in pediatric patients exceeding 90% have been published [15–17]. However, the exact criteria by which patients are selected for NOM has changed over time and continues to be debated [18–20]. In our institution, however, treatment guidelines have not changed over time. All children who are hemodynamically stable and without clinical signs of hollow viscus or diaphragm injury are selected for NOM. Furthermore, when surgical intervention is indicated we prefer to utilize spleen-preserving operative procedures in children. The current study was conducted to determine the outcome of our selective NOM focussed protocol in children with blunt splenic injury. Moreover, we aimed to identify factors associated with persistent hemodynamic instability present in the emergency department that thereby mandated early surgical intervention in children. It is believed that splenic injury grading systems poorly predict the need for surgical intervention for blunt splenic trauma in adults [21]. Nevertheless, given the unique anatomical characteristics of the pediatric spleen, including a relatively thick capsule with enhanced arrangement of elastic capsular fibers. [22, 23], we hypothesized that splenic injury severity is predictive for surgical intervention in children with blunt splenic trauma.

## Material and methods

From our prospectively composed database we included pediatric patients (up to 17 years of age) that presented to our institution with blunt splenic injury over a twelve year period between 01.01.2000–01.01.2012. All pediatric patients with splenic injury that were admitted to the emergency room of our level I pediatric trauma centre were included in this spleen injury registry. Patients who died in the emergency department before diagnostic work-up were excluded. In accordance with our advanced trauma life support (ATLS)-based guidelines [1], all hemodynamically stable patients that do not exhibit symptoms of hollow organ or diaphragm injury are selected for NOM. The determination of a child’s hemodynamic status must be evaluated dynamically with continuous monitoring of vitals, laboratory parameters, skin color, extremity perfusion and neurological status. Furthermore, in our institution the response to weight-adjusted fluid resuscitation dictates therapy and should also be serially evaluated. 

In the presence of hemodynamic shock, a bolus of 20 ml/kg warmed isotonic crystalloid solution is administred. This fluid supplementation regimen has a diagnostic function to examine the patient’s cardiopulmonary status. In the case of an adequate response (*responders*), blood pressure improves and both pulse and respiratory rates drop. In the absence of improved hemodynamics after the first fluid bolus, up to two additional boluses may be provided. Should a hemodynamic response still be absent, the patient should be considered a *non-responder* and emergency intervention is indicated.

Some patients initially respond to fluid resuscitation, but fail to maintain a sustained improvement in hemodynamics. These patients are considered as *transient responders.* Transient responders might require continued fluid resuscitation (and limited packed red blood cells transfusions (pRBC)). If there is no improvement despite the providence of blood products, the patient’s hemodynamic status is defined as inadequately compensated and emergency surgical intervention is indicated as well. Transfusion of large numbers of packed red blood cell transfusions are avoided in our institution as it has been shown that pRBC-transfusions are associated with impaired outcome in critically ill pediatric patients [24]. Should repeated pRBC-transfusions be required to maintain normovolemia and prevent anemia, operative intervention is therefore also indicated.

Hemodynamically compensated patients are selected for nonoperative management, regardless of concomitant injuries, except in the setting of hollow organ and diaphragm involvement. NOM includes initial observation in a monitored intermediate care unit or (pediatric)-Intensive Care Unit (ICU), continuous monitoring of vitals (blood pressure, pulse rate, urine production, temperature. Serial laboratory analysis (initially every 2 h), physical examinations and abdominal sonography (every 4 h). The institutional guidelines utilized, and the physicians managing patients did not change during the study period.

Documented data included patient demographics, Glasgow Coma Scale (GCS), spleen Abbreviated Injury Scale (AIS), Injury Severity Score (ISS), hemodynamic parameters, management and outcome. To analyze the impact of early coagulopathy on outcome, we used the same criteria as Macleod et al. [25]. Coagulopathy was, therefore, defined as the presence of Prothrombin Time (PT) > 14.0 s or Activated Partial Tromboplastin Time (APTT) > 34 s.

Patients were categorized by type of treatment they initially received.

Group I consisted of patients initially treated by nonoperative management and Group II consisted of patients selected for initial operative therapy. Main outcome parameters were failure of nonoperative management (fNOM), complications, length of hospital stay (LOS), length of intensive care unit stay (ICU-stay) and mortality. Splenic Abbreviated Injury Scale (AIS) was determined by using the 2008 version of the Abbreviated Injury scale [26]. Hemodynamic parameters included admission systolic blood pressure (SBP) and admission pulse rate (PR). Failure of NOM (fNOM) was defined as any situation in which a patient was selected for NOM and later required surgical intervention. Complications were tracked in the trauma registry, and all individual charts were reviewed to minimize missing data.

To identify which factors predicted surgical therapy in splenic trauma we used a backward stepwise logistic regression analysis. First, univariable analysis was performed and all factors with a positive *p*-value of less than 0.05 were selected for multivariable analysis. A backward stepwise logit regression analysis was performed and our model was validated by a forward regression analysis.

Statistical analysis was performed using SPSS for Windows 22.0 (Chicago, Illinois). The differences between groups were calculated with Fisher’s Exact Test for ordinal data and Mann-Whitney U test for continuous data. *P*-values less than 0.05 were considered significant.

## Results

A total of 63 pediatric patients sustained blunt splenic injuries during the study period. One patient died on admission and was excluded leaving a total of 62 patients (45 male and 17 female) with a median (Interquartile range, IQR) age of 12 (8–16) included in the study analysis (Table 1).

**Table 1:** Patient characteristics on admission

Age (in years)	12 (8–16)
Gender (M/F)	45/17
SBP (in mmHg)	119 (110–129)
Pulse rate (in bpm)	88 (77–111)
GCS-score	15 (15–15)
Serum Hb (in mmol/L)	7.4 (6.7–8.1)
Serum Ht (in L/L)	0.34 (0.31–0.38)
Thrombocyte count (1 × 10^9^/L)	266 (173–277)
Coagulopathy	20/62
ISS	16 (12–29)
AIS-score spleen	3 (3–4)

All variables are in median (IQR). *Abbreviations*: *SBP* Systolic blood pressure, *bpm* beats per minute, *GCS* Glasgow Coma Score, *Hb* Hemoglobin, *Ht* Hematocrit, *ISS* Injury Severity Score, *AIS* Abbreviated Injury Score

As shown in Table 2 motorcycle accidents accounted for most injuries (*n* = 15). Falls from a height (*n* = 10) and falls from a bicycle (*n* = 7) were the second and third most frequent causes of blunt splenic injury respectively. Median (IQR) ISS was 16 (12–29), and 16 patients had an AIS splenic injury grade greater than 3.

**Table 2:** Mechanism of injury

	*N=*
Motorcycle accident	15
Fall (< 3 m)	10
Bicycle accident	7
Motor vehicle accident	6
Motor vehicle versus bicycle	5
Auto versus pedestrian	4
Other (sports/assault)	15

**Fig. 1:**
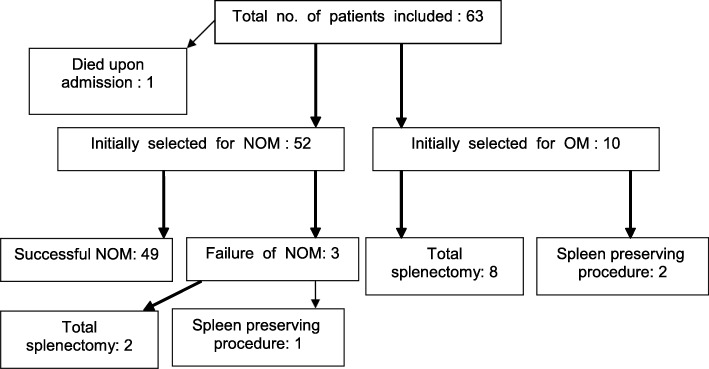
Flowchart

Fifty-two patients (group I) were selected for initial nonoperative therapy and ten patients (group II) underwent direct surgical intervention (Fig. 1). Baseline characteristics of both groups are shown in Table 3. Injury Severity Score was significantly higher in patients that were treated surgically as compared to patients under NOM (36 (23–45) versus 16 (9–18)), *p* = 0.001). The median (IQR) AIS-spleen was also significantly worse in the surgery group  3 (2-4) vs. 4 (4-5), *p* < 0.001.

**Table 3:** Comparison of baseline characteristics: Nonoperative versus operative management in blunt splenic injury

	Nonoperative management *(N = 52)*	Operative management *(N = 10)*	*P*-value
Age (in years)	12 (8–16)	12 (9–16)	0.765^b^
Gender (M/F)	38/14	7/3	1.000^a^
SBP (in mmHg)	117 (110–130)	120 (103–126)	0.898^b^
Pulse rate (in bpm)	88 (77–111)	110 (79–120)	0.255^b^
GCS-score	15 (15–15)	15 (6–15)	0.437^b^
Serum Hb (in mmol/L)	7.5 (6.7–8.1)	7.1 (5.8–7.4)	0.156^b^
Thrombocyte count (×10^9/L)	227 (181–277)	201 (139–273)	0.225^b^
Coagulopathy	14	8	0.062^a^
AIS-score spleen	**3 (2–4)**	**4 (4–5)**	**0.001**^**b**^
ISS	**16 (9–18)**	**36 (23–45)**	**0.001**^**b**^

^a^Fisher’s Exact Test; ^b^Mann Whitney U test. *Abbreviations*: *SBP* +++systolic blood pressure+++, *bpm* +++beats per minute+++ *GCS* Glasgow Coma Score, *Hb* Hemoglobin, *Ht* Hematocrit, *ISS* Injury Severity Score, *AIS* Abbreviated Injury Score. Bold parameters were selected for multivariable analysis (as *P*-value < 0.05)

 Surgical intervention in group II was mandated by our treatment algorithm as these patients exhibited persistent hemodynamic instability despite adequate fluid resuscitation combined with symptoms of an acute abdomen (suggesting ongoing splenic blood loss) in the emergency department. None of these patients had concurrent hollow organ/diaphragm injuries.

Eight patients received a total splenectomy and two patients underwent a spleen preserving procedure by supporting the splenic capsule with a Vicryl mesh (group  II). The median length of stay (IQR) of patients in the surgical group was 12 (5–21) days and median ICU-stay (IQR) was 4 (1–7) days (Table 4).

**Table 4:** Outcome of management for blunt splenic injury

	Nonoperative management *(n = 52)*	Operative management *(n = 10)*	*P*-value
Length of stay (in days)	9 (8–12)	12 (5–21)	0.638^b^
ICU stay (in days)	3 (1–4)	4 (1–7)	0.383^b^
Number of complications	6	3	0.151^b^
Mortality	0	1	0.161^a^

All variables are in median (IQR). ^a^Fisher’s Exact Test, ^b^Mann Whitney U test

One patient from group II developed respiratory insufficiency due to pneumonia. Another patient in this group suffered from an iliac vein thrombosis. This thrombotic complication was probably not a result of post-operative thrombocytosis in splenectomized patients, a phenomenon that occurs frequently following splenectomy. When the thrombotic event occurred, total platelet count was 240,000 cells per μL. The patient had a central femoral vein catheter in situ at the time of thrombosis, which is the most likely explanation for the thrombosis.

Further, a polytrauma patient with an ISS of 57 involved in a motor vehicle accident, died on postoperative day five. This 8-year old patient was hemodynamically stable on admission to the emergency department, however during computed tomography scanning his hemodynamic condition deteriorated. The CT scan revealed that the child had a grade-V splenic injury with a contrast blush. Given his hemodynamic condition an emergency laparotomy was indicated. The spleen was treated with a splenic repair procedure. Intraoperatively an abdominal compartment syndrome with high ventilation requirements developed, and, the abdomen could not be closed. Postoperatively his clinical condition in the pediatric intensive care unit worsened with the additional development of acute respiratory distress syndrome. The patient died after developing Multiple Organ Dysfunction Syndrome on postoperative day five. Seven patients in group II had an uncomplicated clinical course (Table 5).

**Table 5:** Complications in patients treated by nonoperative and operative management

Operative management (n=3)	
Pneumonia	1
Acute Respiratory Distress Syndrome	1
Venous thrombosis	1
Nonoperative management (n=6)	
Pneumonia	1
Urinary tract infection	1
Fever of unknown origin	1
Wound infection	1
Slipped ligature after surgery	1
Acute Respiratory Distress Syndrome	1

Of the 52 patients in the NOM group, three patients later received surgery (fNOM). The development of persistent hemodynamic instability, despite adequate fluid resuscitation, was the indication for surgical intervention in all three patients and hollow organ or diaphragm injuries were not encountered. Failure of NOM occurred in one patient within 24 h after admission, in one patient each at day 3 and day 7. Two patients were treated by splenectomy and one with a spleen preserving procedure. One of these patients received a relaparotomy due to persistent postoperative bleeding. The other two patients had an uncomplicated clinical course. Based on the principle of intention to treat, all patients were analyzed based on their original treatment group. One patient initially selected for nonoperative management who subsequently developed hemodynamic instability, was successfully treated with angio-embolization, thus therefore not requiring surgery and therefore not failing NOM. Other complications encountered in non-operatively treated patients (group I) were pneumonia (*n* = 1), urinary tract infection (*n* = 1) and fever of unknown origin (*n*=1). Another patient had a wound infection of his lower extremity and one patient developed acute respiratory distress syndrome (Table 5).

A comparison of outcomes in both treatment groups is shown in Table 5. Groups did not significantly differ in number of complications. Hospitalization time and ICU-stay were similar between groups as well. Moreover, there was no significant difference in mortality between groups. Mortality was not seen in those patients selected for NOM. To identify predictive factors for hemodynamic instability and subsequent indication for operative therapy a multivariable regression analysis was performed. Univariable analysis (Table 3) demonstrated both ISS (*p* < 0.001) and AIS-spleen (*p* < 0.001) as relevant factors and these variables were therefore selected for multivariable analysis. A stepwise backward logistic regression analysis revealed only severity of splenic injury (AIS-spleen) as an independent predictor for failure of NOM (fNOM). Higher ISS was not statistically significant predictive for operative therapy (Table 6). The backward stepwise logit regression analysis was performed and our model was validated by a forward regression analysis in which comparable results were found.

**Table 6:** Stepwise logit regression analysis for the risk of persistent hemodynamic instability and early operative intervention

Variable	Odds ratio	Lower (95% C.I.)	Upper (95% C.I.)	*p*-value
AIS-spleen	1.117	1.047	1.192	0.001
*Chi*^*2*^ *(df = 6)*	*2.495*	*Sign: 0.869*^a^		

^a^Hosmer and Lemeshowtest for goodness of fit

## Discussion

This study demonstrates that pediatric NOM of splenic injuries:

1. is a safe treatment modality, in well-equipped trauma institutes for *all* hemodynamically compensated children without signs of concomitant hollow organ or diaphragm injuries.
2. is not associated with longer hospitalization times, ICU-stay, or higher complication rates or mortality in children.
3. Further, this study is to the first to demonstrate that pediatric splenic injury severity, rather than other trauma or patient specific admission parameters, predicts persistent hemodynamic instability after trauma and therefore the need for early surgical intervention .

The improved understanding of the crucial role of the spleen in the immune system has resulted in a management shift toward selective nonoperative management for blunt splenic injury [3–5, 10]. This trend was seen in our institution as well. Further, since selection criteria and management protocols were unaltered during the study period, an analysis of outcome and safety in our endeavor to preserve splenic function in children could be performed.

Fifty-two out of 62 patients were treated by NOM and failure occurred in only 3 patients. So, in our study a relatively large number of patients was initially selected for operative intervention [4, 5, 27]. This is most likely due to the relatively high number of severely injured children admitted to our institution. In accordance with prehospital triage guidelines in our region, less severely injured children are admitted to other trauma institutes in our region, whereas major cases of pediatric trauma are preferentially transferred to our hospital.

The failure rate of NOM in pediatric series reported in literature ranges between 2 and 10%, and this is in line with our findings [9–11].

If NOM in our institution proves inadequate, patients are preferably treated with angio-embolization. We consider pediatric angio-embolization as an adjunct to nonoperative management rather than an initial therapeutic option.

Persistently hemodynamically unstable patients underwent emergency explorative laparotomy. When operative intervention is unavoidable, the utilization of spleen saving procedures can contribute significantly to the splenic preservation rate, and in our study these surgical methods were not associated with morbidity or mortality. In our opinion, spleen saving surgery (Vicrylmesh application or splenorrhaphy) may be more successful in children than in adults due to the specific anatomical and mechanical characteristics (thicker capsule and biomechanically optimal arrangement of elastic fibers) of the pediatric spleen as well as more potent tissue healing capacity in children [22, 23]. Splenic preservation should only be attempted if intraoperative hemostasis can be promptly achieved and no signs of continued cardiopulmonary or metabolic deterioration are present. Surgical interventions and treatment decision-making was performed exclusively by pediatric trauma surgeons, which is believed to improve outcome in pediatric splenic trauma [28].

All hemodynamically stable patients and transient responders to resuscitation undergo computed tomography (CT) scanning. In the presence of a contrast blush on CT-scan, patients are considered as candidates for angio-embolization when selected for nonoperative management. However, in the absence of sustained hemodynamic abnormalities, radiological intervention is postponed. Only when the hemodynamic status of patients with a contrast blush on CT deteriorates is embolization indicated. This policy is in line with the literature that shows that in the presence of a contrast blush on CT, pediatric patients can be successfully treated withoutimmediate angio-embolization [29–31], which contrasts with a recent recommendation from Bhullar et al. [16]. In our institution, an interventional radiologist is available 24 h a day and patients with splenic injuries are initially continuously monitored in the pediatric intermediate/intensive care unit. This affords the opportunity to postpone splenic intervention in NOM patients until hemodynamic instability is observed. In our view, these organizational factors are prerequisites for safe nonoperative management in high-grade pediatric splenic injuries. The feasibility of this protocol is supported by the successful outcome of delayed embolization in the single child that developed hemodynamic instability after initial nonoperative management.

As anticipated, no clear differences were encountered in the hemodynamic admission parameters between study groups. We feel, this is at least partly due to the quality and efficiency of pre-hospital care in our region. Resuscitation is initiated as rescue personnel arrive on scene and all severely injured children in our region are transferred to our pediatric level-I-trauma centre. Even low volume fluid resuscitation in children has a relatively large impact on circulating volume. Hemodynamic parameters such as blood pressure, pulse rate, urine production and laboratory parameters may therefore stabilize temporarily even in patients with severe active bleeding. This explains the relatively normal admission parameters of the surgically treated, hemodynamically unstabe patient group II. Moreover, due to enhanced compensatory capacities to initial blood loss in pediatric patients, cardiopulmonary deterioration occurs suddenly and is not preceded by gradually worsening vitals. Consequently, admission hemodynamics as a single entity might not accurately reflect, or underestimate, actual cardiopulmonary status and risks to the injured child. In our view, single initial admission hemodynamic parameters are therefore of minor relevance and hemodynamic changes over time should dictate treatment decisions.

Splenic injury severity is not considered a determining factor in the patient selection process for nonoperative management as per recent literature,no correlation between splenic injury grade and treatment success or safety of NOM was found [32, 33]. In a study from Yang et al. the outcome of NOM for grade IV-V splenic injuries was evaluated. Their study of 42 patients with highgrade splenic injuries showed that NOM was a safe treatment modality. In their study, only one complication occurred and mortality was not seen in high-grade splenic injuries [32]. Furthermore, a study conducted by Jim et al. showed a NOM success rate of 84% in a population of 284 children with high-grade splenic injuries. Patients that failed NOM had similar mortality, length of hospital stay and intensive care unit stay compared with patients initially treated by operative management [34]. In our study a total of 28 patients had splenic injuries with an AIS ≥ 3 and mortality did not occur. Besides, high-grade splenic injury is not an indication for angio-embolization in our institution and our data underlines the safety of this protocol as failure of NOM occurred in just 3 out of 52 patients with no mortality. However, it may be interesting to compare outcome of our cohort with other institutions in which angio-embolization is performed more frequently, for example in the setting of high grade splenic injury or contrast blush. Despite the excellent safety outcome in the current study, outcomes in pediatric bunt splenic injury may potentially benefit from a more liberal utilization of early angio-embolization. To investigate this hypothesis, a (prospective) multicenter study is required.

Interestingly, even though splenic injury severity does not dictate therapy decision making in our institution, the current study reveals that higher splenic injury grades do predict persistent hemodynamic instability and thereby the need for early surgical intervention. To our knowledge, this has not been described before and contrasts with recent studies, including a study by Ardley et al. of 30 pediatric patients and a retrospective multicenter study (which also included rural l trauma centres ) from Adams et al. [35, 36]. We believe that this phenomenon applies only to the pediatric trauma population due to specific anatomical, mechanical and metabolic features of the pediatric spleen [22,23]. A prerequisite for propper injury grading, however, is CT-imaging.  In our opinion, CT-scanning is indicated in all children with potential blunt splenic trauma for the following reasons:

1. as mentioned previously, for splenic injury grading: to determine the risk of persistent hemodynamic instability and estimate the duration of intensive care monitoring. As pediatric splenic injury grading has low inter-radiologist variability, grading is believed to be very reliable [37].
2. to detect a contrast blush, indication for angio-embolization in the setting of delayed hemodynamic instability.
3. to help guide follow-up imaging, make vaccination recommendations and help estimate discharge management [38].

The predictive value of splenic injury severity in pediatric patients with isolated BSI has been underlined by the APSA guidelines as well. These guidelines suggest basing treatment decisions (ICU-stay, hospital stay and imaging) in isolated splenic injuries on the degree of splenic injury [12]. Implementation of grading-based-APSA-guidelines successfully improved outcomes and treatment efficiency of care in children diagnosed with splenic trauma [39]. Adding to APSA-recommendations, the current study demonstrates that splenic injury severity/grade seems to affect treatment decision-making and is predictive for NOM success not only in isolated splenic injuries, but also in patients with multiple injuries. 

In the early years of the NOM-era, surgeons were more restrained in attempting NOM than they are nowadays. Polytrauma, concomitant neurological injury, high grade splenic injury, and significant levels of hemoperitoneum were considered relative contraindications for NOM. However, there is no evidence in the current literature that supports the restrictive use of NOM in the presence of these relative contraindications [4, 12, 20, 31, 32, 34–36, 38].

Due to the anatomical location of the spleen, concomitant injuries are not uncommon in blunt splenic injury. The trend towards NOM of abdominal injuries has led to concerns of missing hollow organ injuries that can increase morbidity and mortality [40]. Our study showed, in line with Miller et al. that no associated injuries were missed by routine diagnostics, and this potential complication should not influence decision-making for NOM [41]. Since less than 1 % of patients with blunt abdominal trauma suffer from relevant hollow viscus injuries, we believe that explorative operative interventions to definitively rule out these injuries is not necessary in the setting of adequate radiologic imaging [42].

After discharge, patients were allowed to return to normal activities. Participation in contact sports was prohibited for a period of 3 months, as complete healing of all grades of splenic injury was confirmed after this period [43, 44]. With respect to follow-up, we are less liberal than the APSA-guidelines for isolated splenic injury because in our opinion tissue healing may be impaired in polytrauma conditions [12]. This is supported by a recent study from Dickinson et al. in which splenic healing time in high-grade injuries seemed to be prolonged (> 8 weeks) [27]. In follow-up, no study patient was readmitted to our institution. 

In line with international vaccination data after splenic trauma, we observed suboptimal vaccination rates in our pediatric population [38, 45]. Our vaccination guidelines after splenic trauma have therefore recently been improved as described in a publication from Spijkerman et al. [38].

## Limitations

The small sample size in this study prevented data stratification. Nevertheless, the study showed that NOM is safe to attempt in the setting of polytrauma and high-grade splenic injury. We further managed to build and utilize a robust logistic regression model to identify predictors for surgical intervention on admission. Unfortunately, we were unable to determine the degree of hemoperitoneum from our database.

## Conclusions

The current study demonstrates that nonoperative management is safe in well-equipped pediatric trauma hospitals for *all* hemodynamically stable pediatric patients without concomitant hollow organ or diaphragm injuries. In the case of persistent hemodynamic instability despite adequate fluid resuscitation, we recommend utilizing spleen preserving surgical treatment options whenever possible. Furthermore, in contrast to the current literature, this study reveals that splenic injury severity does predict persistent hemodynamic instability and the need for early operative intervention in pediatric blunt splenic injury. This study further suggests that initial admission hemodynamic parameters in children are not reliable predictors of the need for surgery in splenic trauma. Therefore, all children, regardless of splenic injury grade should be monitored very closely during the resuscitation phase, even in the setting of normal hemodynamics on admission. Furthermore, patients with more severe higher splenic injuryies are prone to develop early hemodynamic decompensation requiring intervention. Future prospective/multicenter studies should focus on safety of patient selection criteria for nonoperative management. These studies are required to validate the use of splenic AIS in predicting the need for emergency intervention and optimizing decision making in pediatric splenic trauma.

## Data Availability

The datasets used during the current study are available from the corresponding author on reasonable request.

## Abbreviations

AIS: Abbreviated Injury Scale
APSA: American Pediatric Surgical Association
APTT: Activated Partial Thromboplastin Time
ATLS: Advanced Trauma Life Support
BSI: Blunt splenic injuries
CT: Computed tomography
fNOM: Failure of NOM
GCS: Glasgow Coma Scale
ICU: Intensive Care Unit
IQR: Interquartile range
ISS: Injury Severity Score
LOS: Length of hospital stay
NOM: Nonoperative management
OPSI: Overwhelming post splenectomy infection
PR: Pulse rate
pRBC: Packed Red Blood Cell
PT: Prothrombin time
SBP: Systolic blood pressure
